# Beyond Body Condition Score: A Proposed Interspecies Framework for Tissue-by-Tissue Nutritional Assessment in Veterinary Practice

**DOI:** 10.3390/vetsci13090938

**Published:** 2026-09-10

**Authors:** Maria Grazia Cappai

**Affiliations:** 1 Department of Innovation, University of Sassari, via Porto Romano 8, 07026 Olbia, Italy; mgcappai@uniss.it; Tel.: +39-079229444; 2 Nutrition Desk Unit of the Veterinary Teaching Hospital, Department of Veterinary Medicine, University of Sassari, via Vienna n. 2, 07100 Sassari, Italy

**Keywords:** nutritional assessment, physical examination, comparative nutrition, body condition score, nutritional semiotics, interspecies framework, metabolic pathways

## Abstract

In clinical veterinary medicine, the Body Condition Score (BCS) serves as a validated, semi-quantitative estimation of subcutaneous adipose reserves, yet it cannot by itself detect systemic nutrient deficiencies or excesses that do not necessarily imply energy storage. Human clinical dietetics addressed an analogous diagnostic gap through the Nutrition-Focused Physical Examination (NFPE), but veterinary medicine still lacks a unified, multi-species physical examination sequence for nutritional screening. This narrative review synthesizes the comparative and clinical nutrition literature into a proposed, operational interspecies framework for nutritional assessment, designed to complement the BCS and the Muscle Condition Score (MCS) within the existing WSAVA and AAHA nutritional assessment guidelines. The approach sequences a detailed feeding and behavioural anamnesis with a systematic, tissue-by-tissue physical examination of the integument, mucous membranes, skeleton, muscle and joints, and endocrine-metabolic status, supported by metabolic and haematological profiling. Seven synoptic diagnostic tables link observable clinical signs to specific micro- and macronutrient imbalances, the underlying nutritional mechanisms, and the species-specific dependence on key nutrients. Key interspecific determinants are integrated to support differential diagnosis and to caution against uncritical cross-species extrapolation. Each clinical entry is explicitly classified as a primary nutrient deficiency or excess, a nutrition-responsive or nutrition-related metabolic disease, a disease causing secondary malnutrition, or a management or behavioural determinant of intake, and each table states the principal non-nutritional differentials and the confirmatory test required, so that the tables function as screening prompts. The resulting semiotic methodology is intended to extend nutritional assessment beyond fatness (assessed on extension of explorable sub-cutaneous adipose tissue) towards whole-body nourishment. It is presented as a proposed framework and not as a validated instrument, and it requires prospective validation before any routine clinical adoption across animal species. The supporting evidence is substantially stronger for dogs, cats, horses, and ruminants than for avian, reptile, and small exotic mammal species, for which the present extension is a proof of concept requiring dedicated species-specific validation.

## Simple Summary

Evaluating the nutritional state of a patient is part of the physical examination, from fatness assessment through the Body Condition Score (BCS), or lean masses through the Muscular Condition Score (MCS). Nutrient deficiencies or excesses may nonetheless affect other examinable organs. While human medicine uses comprehensive physical exams to detect malnutrition, practical veterinary nutrition may still require such a holistic approach for complete nutritional screening. This paper proposes a practical, step-by-step clinical nutrition examination framework for veterinarians working with companion, livestock, and exotic animals. Through systematic evaluation of the nutritional state of individually examinable tissues, and in view of metabolic differences among species, this framework is intended to provide a structured basis for diagnostic reasoning, linking visible physical signs to possible underlying nutritional imbalances, so as to appraise whole-body nourishment and support animal care. The framework is designed to be used together with, and to complement, body and muscle condition scoring, and it is presented as a proposal.

## 1. Introduction

In routine veterinary clinical practice, the Body Condition Score (BCS) is widely adopted for the nutritional assessment of patients [1]. The BCS system provides a validated, semi-quantitative estimation of sub-cutaneous adipose tissue (SAT) depots through visual inspection and palpation across key anatomical regions [1]. While the BCS effectively estimates fat storage, it is necessarily limited when a holistic assessment of the animal’s nutritional condition, understood as its overall state of nourishment, is required [2]. The BCS therefore remains an essential and non-negotiable component of every nutritional assessment; the argument developed here is that it is necessary but not sufficient, and that it should be complemented by tissue-specific semiotics and laboratory analyses for overall nutritional state assessment [1,3].

Malnutrition encompasses a broad spectrum of pathological states resulting from relative or absolute deficiencies, excesses, or imbalances of essential macro- and micronutrients [2], and is therefore not limited to the energy stored as fat. Subclinical or clinical nutrient imbalances frequently induce organ-, tissue-, and cellular-level alterations in animals exhibiting a normal body weight or a target BCS according to size [2]. Early-stage nutritional disorders that impair systemic homeostasis, immune competence (for example, epithelial barrier failure and secondary bacterial infection of hypovitaminosis A), dermatological integrity, and musculoskeletal health (for example, nutritional myodegeneration and metabolic bone disease) should also be accounted for during a nutrition-focused examination by the veterinarian.

In human clinical dietetics, the establishment of the Nutrition-Focused Physical Examination (NFPE) produced an integrated approach designed to detect clinical signs of malnutrition and nutrient-specific deficiency or toxicity syndromes; the underlying competency tool has since progressed through item generation, Delphi-based content and face validation, and formal inter-rater and intra-rater reliability testing among registered dietitians [2,4,5]. In veterinary medicine, the World Small Animal Veterinary Association (WSAVA) and the American Animal Hospital Association (AAHA) introduced the Nutritional Assessment Guidelines, organizing clinical screening around the “Circle of Nutrition” [1]. These consensus guidelines represent the current standard of care, and the present work is built upon them rather than offered as an alternative to them: the WSAVA “Circle of Nutrition” and the 2021 AAHA Nutrition and Weight Management Guidelines already define screening, individualized planning, and client communication comprehensively, and both are endorsed here without reservation [1,3]. Their declared scope, however, is companion animals (dogs and cats), and their physical-examination component is centred on Body Condition Score and Muscle Condition Score (MCS), that is, on the adipose and lean-mass compartments [1,3].

However, an ordered, tissue-by-tissue examination of the remaining accessible compartments (integument and adnexa, mucosae and eye, bone, muscle and joint, oral cavity, and digestive and urinary output) needs to be taken into account in veterinary nutrition-focused assessment. An explicit index of the species-specific metabolic constraints that determine which nutrient imbalances are physiologically plausible in a given patient should be considered with adequate knowledge of animal requirements and physiology. Finally, the assignment of every clinical entry to an explicit aetiological category may be of help in the determination of the role of species-specific nutritional care and support. The first of these is already established practice in human clinical dietetics through the NFPE but has no veterinary equivalent; the second has no counterpart in human medicine at all, which of course addresses a single species. It is this second element that makes the sequence proposed here interspecies rather than species-specific: the order of the examination is common to all taxa, whereas the nutrients screened at each step are dictated by the metabolic traits of the species under examination. The contribution proposed here is therefore additive and complementary, not substitutive. The same gap has been identified independently for ruminants, for which a framework for nutritional assessment in compromised subjects has recently been proposed, integrating signalment, health interview, environmental examination and nutrition-focused clinical assessment, and providing ruminant-specific instruments such as perineal faecal soiling scoring, rumen fill scoring, and a muscle scoring system independent of BCS [6], together with a companion narrative review of clinical nutrition in the same population [7]. That work is specifically related to ruminants and adapts instruments developed for small animals and equids to a single production class. The present framework is constructed across different animal species and is indexed to the metabolic constraints that differentiate them. An operational, interspecies physical examination sequence capable of guiding the clinician through a tissue-by-tissue assessment of nourishment, while simultaneously accounting for the metabolic traits that make a given species dependent on a given nutrient—for example, the feline requirement for preformed vitamin A, niacin, and arachidonic acid, or the canine and feline inability to synthesize vitamin D3 in the skin—has not, to date, been assembled into a single instrument [2]. This paper proposes an integrated, interspecies methodological framework conceived for eventual use within routine clinical consultations. It is presented at the stage of conceptual development, and it is offered neither as a validated clinical protocol nor as an instrument already suitable for routine, unsupervised clinical use. By combining dietary anamnesis, tissue-specific inspection, metabolic profiling, and comparative physiological principles, this framework is intended to bridge the gap between basic comparative nutrition and daily veterinary clinical practice [2].

## 2. Materials and Methods

### 2.1. Literature Search Methodology

This narrative review aimed to construct a clinically ordered examination sequence spanning taxa, nutrient classes, and organ systems, for which no single answerable PICO-type question exists and for which no pooled effect estimate can meaningfully be computed. Since a PRISMA-governed synthesis with formal risk-of-bias grading is designed for a different object of enquiry, the search strategy and the source-selection procedure adopted for the present narrative synthesis are set out below. The conceptual framework and the diagnostic content of Table 1, Table 2, Table 3, Table 4, Table 5, Table 6, Table 7 and Table 8 were developed through a structured search of the veterinary and comparative-nutrition literature indexed in PubMed/MEDLINE, Scopus, and the Web of Science Core Collection, from database inception to the final search date of 10 March 2026. No language restriction was applied at the search stage; records not retrievable in English were not assessed. Search strings combined the terms “nutrition-focused physical examination”, “body condition score”, “muscle condition score”, and “comparative nutrition” with species designations (canine, feline, equine, bovine, ovine, caprine, porcine, avian, reptile, and lagomorph) and with lesion- or nutrient-specific terms (e.g., “zinc-responsive dermatosis”, “white muscle disease”, “enzootic calcinosis”, “equine metabolic syndrome”, and “perosis”). Eligibility criteria were defined *a priori*. Records were eligible if they were peer-reviewed journal articles (original research, case series, or reviews), authoritative consensus guidelines, or standard reference texts; if they reported a clinical sign, lesion, biomarker, or metabolic trait attributable to a nutrient deficiency, a nutrient excess, or a species-specific metabolic constraint; and if the species concerned was a domestic, companion, livestock, or exotic companion vertebrate. Records were excluded if they were conference abstracts without retrievable methods, non-peer-reviewed material, in vitro or rodent-model studies without stated relevance to the target species, or therapeutic trials without a diagnostic or semiotic component. Retrieved records were examined in two stages, first by title and abstract and subsequently in full text, and the reference lists of retrieved reviews and standard reference texts were hand-searched for further eligible sources (citation chaining). Consistent with a narrative design, sources were selected purposively for clinical relevance to the examination sequence rather than through exhaustive enumerative screening; the number of records retrieved and excluded at each stage was consequently not recorded, and no PRISMA flow diagram is presented. In addition, screening and selection were performed by a single author, so no inter-screener agreement statistic could be computed. The evidential basis of every table entry is qualified in the notes accompanying each table, which state the species in which the finding is documented, the category to which the entry belongs, the principal non-nutritional differentials, and the confirmatory test required.

### 2.2. Methodological Approach and Clinical Sequence

**Figure 1:**
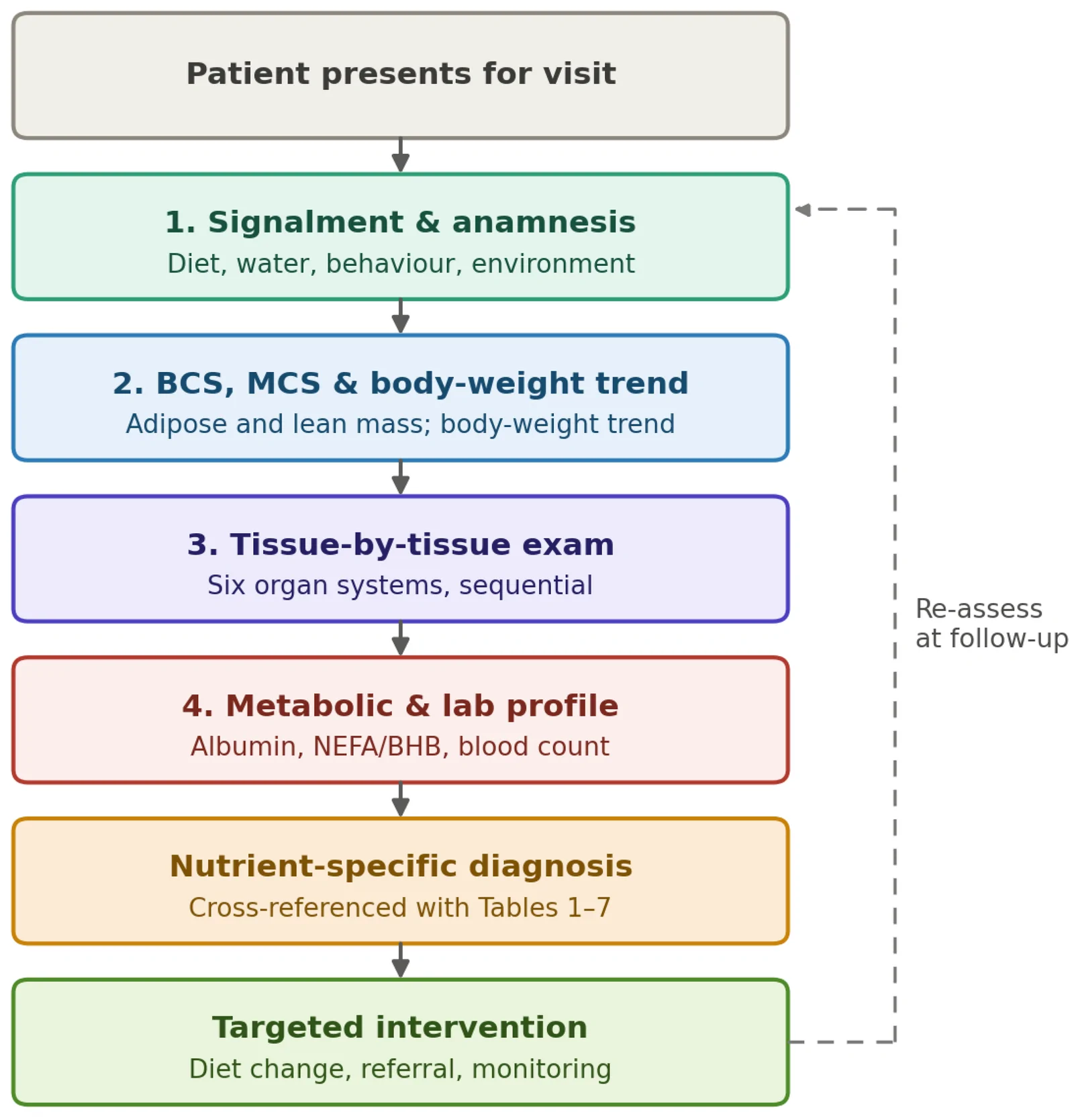
Operational algorithm of the interspecies nutritional physical examination, from clinical presentation to targeted intervention and follow-up reassessment.

The operational sequence comprises four steps: (i) General Signalment and Feeding Anamnesis [1,3]; (ii) Body Condition Score and Muscle Condition Score, performed first and in every patient as the established quantitative anchor of the assessment [1,3,8,9,10]; (iii) Special Tissue-by-Tissue Physical Examination, proceeding from inspection to palpation, then to the explicit exclusion of non-nutritional aetiologies, and only thereafter to attribution to a specific deficiency or excess [2]; and (iv) Metabolic and Haematological Assessment [11,12]. Steps (ii) and (iii) are complementary and neither substitutes for the other: BCS and MCS quantify the adipose and lean compartments, whereas the tissue-by-tissue examination screens compartments that BCS and MCS do not account for. A consolidated operational checklist, including the minimum laboratory database by species, referral triggers, and reassessment intervals, is given in Table 8. Throughout, the sequence is presented as a proposed screening framework intended to complement, and never to replace, the existing WSAVA and AAHA guidelines; it has not undergone psychometric validation, and none of its individual steps should be read as a validated diagnostic criterion. Figure 1 summarizes the operational algorithm followed during the clinical visit, including its feedback loop to follow-up reassessment.

### 2.3. Classification of Entries with Four Aetiological Categories

A recurrent difficulty in veterinary nutritional assessment is that clinically important, nutrition-relevant findings do not all share the same aetiological status. A zinc-responsive dermatosis, a case of feline diabetic polyneuropathy, a painful periodontal disease, and competition at a shared food bowl are all legitimate objects of a nutritional consultation, but they are not the same kind of entity. Each clinical entry in Table 1, Table 2, Table 3, Table 4, Table 5, Table 6 and Table 7, that is, each describing a lesion, a clinical sign, or a disease entity, is therefore assigned to one of four explicit aetiological categories, indicated in square brackets in the first column and in the table notes. A minority of rows are not clinical entries in this sense and consequently carry no category code: these comprise physiological comparator rows, which describe normal species-specific metabolic traits included to constrain cross-species inference rather than to denote pathology (Table 5), and monitoring parameters, which are quantitative indicators tracked over time rather than diagnoses in themselves (notably faecal score and urine specific gravity in Table 6, and every analyte in Table 7). A physiological trait is not a finding to be corrected, and a monitoring parameter is interpretable only from its serial pattern.

*Category A—Primary nutrient deficiency or excess*. The clinical sign is caused directly by inadequate or excessive intake, absorption, or utilization of a specific nutrient, and resolves upon correction of that nutrient. Examples include zinc-responsive dermatosis, nutritional myodegeneration from selenium and vitamin E deficiency, hypovitaminosis A, and ovine chronic copper toxicity.

*Category B—Nutrition-responsive or nutrition-related metabolic disease*. The disorder is not caused by a nutrient deficiency or excess, but nutrition is a dominant modifiable risk factor, a principal instrument of management, or both. Diagnosis rests on disease-specific testing rather than on nutritional semiotics. This category comprises periparturient hypocalcaemia and eclampsia, feline diabetes mellitus and its polyneuropathy, equine metabolic syndrome, feline hepatic lipidosis, and canine COMMD1-associated copper storage disease.

*Category C—Disease causing secondary malnutrition*. The primary lesion is not nutritional, but it impairs prehension, mastication, digestion, absorption, or retention, and thereby produces malnutrition as a consequence. Recognition matters because correcting the diet without correcting the underlying disease would fail to resolve the clinical problem. Examples include periodontal disease and feline chronic gingivostomatitis, equine dental malocclusion, chronic enteropathy, and protein-losing nephropathy.

*Category D—Management, behavioural, and interventional determinants of intake*. Neither a nutrient disorder nor a disease, but a management, environmental, behavioural, or therapeutic factor that determines what the animal actually consumes, or an intervention whose evidence base must be judged on efficacy rather than on deficiency. Examples include intraspecific competition at feeding points, psychogenic dysorexia, and interrupted caecotrophy in rabbits.

The four categories are not mutually exclusive in every case, and dual assignment is applied where clinical evaluation warrants it; ovine copper toxicity, for instance, is a Category A intoxication, whereas the canine COMMD1 phenotype sharing the same target organ is Category B. The purpose of the classification is not taxonomic but diagnostic: it requires the clinician to establish, before intervening, whether the finding will resolve through correction of a nutrient, treatment of a disease, removal of an obstacle to intake, or modification of management.

## 3. Results

### 3.1. General Signalment and Dietary Anamnesis

The preliminary stage establishes the medical and management context required for clinical interpretation [1]:

Signalment: Species, breed/lineage, age category (juvenile/growing, adult, or senior/geriatric), sex, and reproductive or productive status (maintenance, gestation, lactation, growth, physical work, or meat/milk/fibre production) [1].

Feeding Anamnesis: Quantitative estimation of daily Dry Matter Intake (DMI) and energy requirements relative to body size, calculated according to the species’ metabolic weight (BW^0.75^); daily water requirements against actual intake; diet composition (feed type, quantity, and nutrient/energy provision, together with feeding practices) [1]. Where owners cannot supply quantitative intake data, which is common in companion-animal practice, practical information should be recorded instead, including the product label and guaranteed analysis, the measuring device actually used (cup, scoop, or scale), the number of meals offered per day, the brand and daily number of treats, chews, and, in the case of household animals, table foods, and any change in diet within the preceding three months [1,3]. A complete medication and supplement history must also be obtained, including anthelmintics, corticosteroids, and over-the-counter mineral or vitamin products, together with reproductive output (parity, litter size, and stage of lactation) and, in livestock, the current production level, since each of these modifies nutrient requirement, voluntary intake, or nutrient handling [3].

Clinical Semiotics: Systematic recording of associated clinical signs, including ptyalism, borborygmi, dysorexia, pica, and feeding-related stereotypic behaviours [1].

Housing and Environmental Context: Evaluation of group-housing dynamics, intraspecific competition, interspecific anxiety or aggression, and physical access restrictions to feeding/watering stations; these management variables must be evaluated independently of diet formulation [1].

### 3.2. Tissue-by-Tissue Nutritional Examination

#### 3.2.1. Integument and Adnexa

The integumentary system (skin, hair, wool, feathers, hooves, and claws) is an accessible early indicator of metabolic and nutritional status owing to its high cellular turnover rate and substantial protein and micronutrient demands [13]. Visual evaluation assesses coat or plumage coverage, symmetry of alopecia, hair lustre, and pigment integrity (discoloration or achromotrichia) [14]. Palpation assesses skin thickness, elasticity, anchorage, scaling, seborrhoea, and follicular hyperkeratosis [15]. Several nutrient classes can produce overlapping cutaneous signs, including trace elements, amino acids, fat-soluble vitamins, and water-soluble vitamins; essential fatty acids and the ω-6 and ω-3 series are considered separately below. Because every one of these signs is non-specific, ectoparasitism, dermatophytosis, allergic and endocrine dermatoses, superficial pyoderma, and immune-mediated disease must be excluded before any cutaneous change is attributed to nutritional or nutrition-associated factors.

Copper (Cu): A required cofactor for tyrosinase, the rate-limiting enzyme in melanin synthesis [14]. Copper deficiency presents as achromotrichia, classically manifesting as periocular pigment loss in ruminants and “steely wool” in sheep [14]. Chronic dietary copper excess causes hepatic accumulation leading to sudden intravascular haemolytic crises in sheep and constitutes a true nutritional intoxication. This must be distinguished conceptually from canine copper-storage hepatopathy associated with COMMD1 mutation, which is a monogenic defect of biliary copper excretion in which diet is a modifying rather than a causal factor [16]. The two conditions share a target organ but not an aetiological category: only the ovine form is a primary nutritional excess, whereas the canine form is a genetic disorder in which dietary copper restriction is mandatory.

Zinc (Zn): Essential for keratinocyte proliferation and differentiation [17]. Zinc-responsive dermatosis presents as symmetrical parakeratotic scaling, erythema, and crusting around mucocutaneous junctions and pressure points, well characterized in dogs [17,18], with analogous zinc-responsive parakeratotic lesions also classically described in swine, calves, and goats. High dietary calcium or phytate impairs zinc absorption in monogastrics [19]. The cutaneous presentation is not specific: sarcoptic and demodectic mange, dermatophytosis, cutaneous adverse food reaction, atopic dermatitis, superficial pyoderma, and endocrine dermatoses (hypothyroidism and hyperadrenocorticism) must be excluded first. Confirmation rests on skin biopsy demonstrating diffuse parakeratotic hyperkeratosis together with clinical response to zinc supplementation, rather than on serum zinc concentration, which is a poor index of status and is depressed non-specifically by inflammation.

Biotin (Vitamin B7): An essential coenzyme for carboxylase enzymes involved in fatty-acid synthesis, gluconeogenesis, and amino-acid metabolism. Biotin plays a crucial role in keratin synthesis, cell differentiation, and maintenance of the epidermal lipid barrier [13]. Deficiency leads to dry, desquamative dermatitis, diffuse alopecia, skin crusting, and compromised hoof, claw, or footpad integrity (e.g., hoof-wall cracking and sole lesions in horses, swine, and ruminants) [13]. Antinutritional factors should also be considered in particular cases. Ingestion of raw egg white containing active avidin, a glycoprotein that binds biotin with very high affinity, can prevent intestinal absorption; this is, however, an uncommon and largely historical cause in contemporary practice, relevant chiefly to unbalanced diets or not meeting augmented requirements, whereas prolonged antimicrobial suppression of intestinal microbial biotin synthesis is the more frequent modern contributor [13].

Niacin and Tryptophan: In dogs, swine, and humans, tryptophan can be converted de novo to niacin via the kynurenine pathway. The historical demonstration that canine “black tongue” and human pellagra share a common nutritional cause [20] is cited here for priority only; the biochemical basis of the pathway, and of its interspecific differences, is established in the modern comparative literature [21,22]. Deficiency manifests as oral ulceration and dark tongue (“black tongue” in dogs) [20]. Conversely, cats possess high activity of picolinic carboxylase, which diverts tryptophan intermediates away from niacin synthesis, rendering them strictly dependent on preformed dietary niacin [21,22]. Clinically, oral ulceration is far more frequently infectious, immune-mediated, uraemic, or neoplastic than nutritional; nutritional niacin deficiency should therefore be considered only after those causes have been excluded and a plausible dietary history has been established.

Vitamin A: Essential for epithelial cell differentiation [15]. Deficiency leads to follicular hyperkeratosis and squamous metaplasia [15]. The antagonistic interaction between vitamin A and vitamin D at the level of skeletal metabolism is considered further below.

**Table 1:** Synoptic screening table for nutritionally relevant integumentary alterations.

Nutrient	Clinical Sign/Lesion	Mechanism (Species Specific)	Literature
Copper (Deficiency)	Achromotrichia (periocular pigment loss), “steely wool”	Impaired tyrosinase cofactor activity; earliest clinical sign in cattle and sheep	Van Ryssen & Webb, 2026 [14]
Copper (Excess) [A/B]	Haemolytic crisis (ruminants); chronic hepatitis (canine)	Hepatic saturation leading to massive Cu release; sheep highly sensitive, dogs (COMMD1 mutation)	Van Ryssen & Webb, 2026 [14]; Van De Sluis et al., 2002 [16]
Zinc (Deficiency)	Symmetrical parakeratotic scaling, alopecia, crusting	Impaired keratinocyte proliferation; documented in dogs; analogous parakeratosis described in pigs, calves, and goats	Colombini, 1999 [17]; Colombini & Dunstan, 1997 [18]
Biotin (Deficiency)	Dry desquamative dermatitis, alopecia, skin crusting, hoof/claw wall cracking	Impaired carboxylase activity and keratin synthesis; compromised epidermal lipid barrier; horses, pigs, cattle, and dogs; induced by raw egg-white avidin	Hensel, 2010 [13]
Vitamin A (Deficiency)	Follicular hyperkeratosis, dull coat, dry scaling	Disrupted keratinocyte differentiation and epithelial maintenance; documented in dogs	Ihrke & Goldschmidt, 1983 [15]
Niacin/Tryptophan	“Black tongue”, oral ulceration, crusting dermatitis	Defective NAD/NADP synthesis; cats require preformed niacin due to high picolinic carboxylase activity	Anonymous, 1928 [20]

Footnotes to Table 1. Category codes: [A] primary nutrient deficiency or excess; [B] nutrition-related metabolic disease; [C] disease causing secondary malnutrition; [D] management, behavioural or interventional factor. All rows are Category A except copper excess, which is [A] in sheep (dietary intoxication) and [B] in dogs (COMMD1-associated storage disease). These signs are screening prompts, not diagnostic proof, which requires clinician evaluation. Principal non-nutritional differentials to exclude for all cutaneous entries: ectoparasitism (sarcoptic and demodectic mange, lice, and Cheyletiella), dermatophytosis, superficial bacterial pyoderma, Malassezia dermatitis, cutaneous adverse food reaction and atopic dermatitis, endocrine dermatoses (hypothyroidism, hyperadrenocorticism, and sex-hormone imbalance), immune-mediated disease (pemphigus foliaceus and cutaneous lupus), and epitheliotropic lymphoma. Confirmatory tests: skin scrapings, trichogram, fungal culture or PCR, cytology, endocrine testing, and skin biopsy for histopathology; zinc-responsive dermatosis is confirmed by biopsy plus response to supplementation, not by serum zinc; copper status by hepatic copper quantification, not by serum copper; vitamin A-responsive dermatosis by biopsy plus response to retinoid therapy. Abbreviations: Cu, copper; Zn, zinc; NAD/NADP, nicotinamide adenine dinucleotide (phosphate); COMMD1, copper metabolism domain containing 1.

#### 3.2.2. Mucous Membranes and Ocular System

Mucous membranes (oral, ocular/conjunctival, and vaginal) provide direct clinical insight into peripheral perfusion, epithelial maturation, and haematological health [12].

Interspecies Synthetic Capacities (Vitamin C): The ability to synthesize ascorbic acid (vitamin C) de novo from D-glucose was lost in specific phylogenetic lineages through inactivating mutations in the gene encoding L-gulonolactone oxidase (GLO) [23]. An obligate dietary requirement for vitamin C is restricted to teleost fish, anthropoid primates, guinea pigs (*Cavia porcellus*), and certain bats and passerine birds [23]. Dogs, cats, horses, swine, and ruminants possess functional hepatic or renal GLO activity and synthesize adequate endogenous vitamin C [23]. Reptiles are not uniform in this respect: renal GLO activity has been documented in several squamate and chelonian lineages, and no obligate dietary requirement has been established for the reptile species commonly kept in captivity. Ascorbate is therefore not screened as a routine deficiency candidate in the reptile subsection below, which focuses instead on vitamin D3 status and the Ca:P ratio [23].

Vitamin A and Retinoid Metabolism: Herbivores efficiently convert dietary β-carotene into active retinol. Cats, as obligate carnivores, lack intestinal β-carotene 15,15′-dioxygenase activity and require preformed vitamin A (retinyl esters) from animal tissue, an idiosyncratic consequence of their evolutionary dietary specialization [21]. Deficiency leads to xerophthalmia, conjunctival keratinization, loss of goblet cells, and nyctalopia. Congenital hypovitaminosis A in calves results in decreased eyesight and optic-nerve constriction, frequently compounded by secondary bacterial infection of the gastrointestinal mucosa [24]. In pigs, β-carotene conversion to retinol is functional but of limited efficiency. The converse situation, in which retinol status is modulated by a non-dietary demand, is illustrated by the albino Asinara donkey, in which circulating retinol fluctuates with photoperiod and appears to be mobilized from body stores as an alternative photoprotective strategy where melanin is absent [25]; species-specific and even breed-specific physiological demand, and not dietary supply alone, may therefore determine circulating vitamin A concentrations. Swine are conventionally regarded as physiologically intermediate between obligate carnivores, which lack the pathway, and strict herbivores, in which it operates efficiently, with conversion efficiency lower than in ruminants, horses, or poultry [19]. This intermediate position is a practical generalization drawn from comparative requirement data rather than from a directly measured enzymatic ranking, and it should be read with that qualification. Because β-carotene degrades rapidly during feed storage and heat processing, diet fortification with synthetic preformed vitamin A (e.g., retinyl acetate or palmitate) is routinely required to prevent hypovitaminosis A in swine, which presents clinically as reproductive failure, incoordination, and epithelial squamous metaplasia [19].

**Table 2:** Synoptic screening table for nutritionally relevant mucous membrane and ocular alterations.

Nutrient	Clinical Sign/Lesion	Mechanism (Species Specifics)	Literature
Vitamin A	Xerophthalmia, conjunctival keratinization, night blindness	Loss of epithelial mucin secretion; documented in cattle, swine, and poultry; congenital form in calves	He et al., 2012 [24]
Vitamin C	Scurvy, mucosal haemorrhages, impaired wound healing	Inability to synthesize ascorbate due to GLO gene loss; teleost fish, primates, guinea pigs, and bats	Drouin et al., 2011 [23]
Iron/B-Vitamins (B12, Folate)	Mucosal pallor, glossitis, cheilitis	Secondary to nutritional normocytic/microcytic or macrocytic anaemia; confirmed via complete blood count	Kaneko et al., 2008 [12]

Footnotes to Table 2. All rows are Category A, but each requires laboratory confirmation and none is diagnostic on inspection alone. Principal non-nutritional differentials: for mucosal pallor, blood loss of any cause, haemolysis (immune-mediated, oxidative, or haemoparasitic), anaemia of inflammatory disease, chronic kidney disease, and bone-marrow disorders; for ocular surface change, keratoconjunctivitis sicca, infectious keratoconjunctivitis, trauma, and environmental irritants; for glossitis and cheilitis, infectious, uraemic, immune-mediated, and neoplastic causes. Confirmatory tests: complete blood count with erythrocyte indices and reticulocyte count, blood smear, serum iron and ferritin, serum cobalamin and folate, faecal occult blood, and serum or hepatic retinol; Schirmer tear test and fluorescein staining for the ocular surface. Iron deficiency anaemia in an adult animal indicates chronic blood loss until proven otherwise. Abbreviations: GLO, L-gulonolactone oxidase; MCV, mean corpuscular volume; MCHC, mean corpuscular haemoglobin concentration; CBC, complete blood count.

#### 3.2.3. Musculoskeletal System—Bone and Mineral Homeostasis

Skeletal proportions, bone density, and growth-plate integrity must be evaluated relative to the animal’s age, growth rate, and reproductive or productive stage [26].

Calcium, Phosphorus, and Vitamin D Interrelationships: The physiological Ca:P ratio must be maintained between 1:1 and 2:1 in most mammals [26]. Excess dietary phosphorus inhibits intestinal calcium absorption, triggering secondary nutritional hyperparathyroidism and fibrous osteodystrophy [26]. The reported frequency of nutritional secondary hyperparathyroidism and fibrous osteodystrophy differs between species: the classical syndrome is most often described in horses, swine, dogs, cats, New World primates, and reptiles, and is reported less frequently in sheep and cattle [26]. This difference, however, must be interpreted with caution, since it reflects the observed frequency of clinical cases under prevailing management conditions, in which forage-based ruminant rations are rarely inverted in Ca:P, rather than a demonstrated difference in the parathyroid response itself. Ruminants are not immune, and the syndrome does occur in cattle and small ruminants fed grain-heavy, low-calcium rations [26,27]. Conversely, the nutritional diagnosis must not be reached by default. Skeletal fragility with pathological fracture may equally reflect a primary endocrine defect, as in a case of underlying primary hypoparathyroidism in an adult spayed bitch in which a pelvic fracture and seizures brought a severe, previously unrecognized hypocalcaemia to clinical attention [28]; ionized calcium and parathyroid hormone, and not the dietary history alone, distinguish the two.

Cutaneous Vitamin D3 Synthesis: Endogenous synthesis of vitamin D3 via solar UV-B irradiation of 7-dehydrocholesterol in the epidermis is highly efficient in ruminants, horses, and swine [26]. Conversely, dogs and cats display very low cutaneous 7-dehydrocholesterol-Δ7-reductase turnover, which channels the precursor toward cholesterol synthesis rather than pre-vitamin D3, rendering them largely reliant on dietary vitamin D3 regardless of sun exposure [29]. New World camelids kept at low altitude or in northern latitudes are highly prone to vitamin D-deficiency rickets owing to their dense fibre coats and reduced solar exposure [26].

Enzootic Vitamin D Hypervitaminosis (Enzootic Calcinosis): Chronic ingestion of calcinogenic plants containing active vitamin D analogues (e.g., 1,25-dihydroxycholecalciferol glycosides found in Solanum glaucophyllum, Trisetum flavescens, or Cestrum diurnum) induces severe systemic vitamin D toxicity [30,31]. Unregulated intestinal absorption of calcium and phosphorus leads to persistent hypercalcaemia and hyperphosphataemia, resulting in extensive metastatic calcification of soft tissues—particularly large blood vessels, heart valves, tendons, and the lungs. Affected ruminants and horses present with progressive weight loss, severe stiffness, shifting lameness, an arched back, and cardiovascular insufficiency [30,31].

Eclampsia and Acute Periparturient Hypocalcaemia (Puerperal Tetany/Milk Fever): A sudden, massive drainage of blood calcium into colostrum and milk during late pregnancy or early lactation can exceed the mobilization capacity of parathyroid hormone (PTH) and bone-resorption mechanisms. The clinical presentation varies markedly across species according to motor end-plate physiology. In small-breed dogs (puerperal tetany/eclampsia), acute hypocalcaemia increases neuromuscular membrane excitability, presenting as muscle tremors, restlessness, panting, severe muscular tetany, hyperthermia, and seizures, typically 1–4 weeks postpartum [32]. In dairy cattle (parturient paresis/milk fever) and small ruminants, hypocalcaemia impairs acetylcholine release at the neuromuscular junction, leading to progressive muscle weakness, flaccid paralysis, sternal or lateral recumbency, and coma around the time of parturition [27,32]. Both syndromes are periparturient metabolic diseases with major nutritional risk factors rather than primary dietary calcium deficiencies, and they are classified accordingly as Category B: the precipitating event is the abrupt lactational calcium drain, while diet, and in particular the prepartum dietary cation–anion difference, modulates risk and forms the basis of prevention [27].

Hypervitaminosis A and Manganese-Dependent Perosis: Hypervitaminosis A antagonizes vitamin D absorption and utilization, resulting in periosteal exostoses, cervical spondylosis, and accelerated epiphyseal closure in growing animals [26]. In cats, this is a chronic, cumulative intoxication requiring months to years of a liver-predominant or liver-supplemented diet; it is not the consequence of an occasional dietary error, and the history should establish habitual, long-term liver feeding before the diagnosis is entertained [26]. A distinct condition, particularly evident in poultry, results from manganese (Mn) deficiency: in avian species, manganese is a critical cofactor for the glycosyltransferases involved in chondroitin-sulfate synthesis during cartilage-matrix formation. Manganese deficiency causes perosis (“slipped tendon”), characterized by enlargement and malformation of the hock joint, flattening of the condyles, and lateral slippage of the gastrocnemius tendon [33]. A distinct plantigrade postural change of neuropathic—rather than skeletal—origin, involving the hock rather than the epiphysis, is described below for diabetic cats.

**Table 3:** Synoptic screening table for nutritionally relevant bone and mineral alterations.

Nutrient/Condition	Clinical Sign/Lesion	Mechanism (Species Specific)	Literature
Vitamin D/Ca/P Imbalance	Rickets, osteomalacia, fibrous osteodystrophy	Dogs/cats have minimal cutaneous D3 synthesis; horses comparatively resistant to rickets; camelids at high risk outside native altitude; fibrous osteodystrophy: horses, pigs, dogs, cats, reptiles > ruminants	Uhl, 2018 [26]; Hazewinkel & Tryfonidou, 2002 [29]
Enzootic Vitamin D Toxicity (Calcinogenic Plants)	Metastatic vascular and soft-tissue calcification, stiff gait, lameness, chronic wasting	Ingestion of 1,25(OH)2D3 glycosides (*S. glaucophyllum* and *T. flavescens*); severe hypercalcaemia/hyperphosphataemia causing tissue calcification in cattle, sheep, and horses	Machado et al., 2020 [30]; Mello, 2003 [31]
Acute Hypocalcaemia (Eclampsia/Milk Fever) [B]	Tetany, seizures, hyperthermia (canine eclampsia); flaccid paralysis, sternal recumbency (bovine milk fever)	Rapid calcium depletion via lactation exceeding PTH-mediated bone resorption; small-breed dogs (puerperal tetany) vs. high-producing dairy cattle (parturient paresis)	Drobatz & Casey, 2000 [32]; Goff, 2014 [27]
Vitamin A (Excess)	Cervical spondylosis, periosteal exostoses	Excessive osteoblast activation and cartilage damage; common in cats fed all-liver diets	Uhl, 2018 [26]
Manganese (Mn)	Perosis (“slipped tendon”), enlarged/malformed hock joints	Impaired glycosaminoglycan synthesis in epiphyseal cartilage via glycosyltransferase cofactor deficiency; specific to poultry, ratites, and other avian species	Lilburn, 2021 [33]

Notes to Table 3. Category codes: vitamin D/Ca/P imbalance, enzootic vitamin D toxicity, vitamin A excess and manganese deficiency are Category A; acute hypocalcaemia (eclampsia, milk fever) is Category B, a periparturient metabolic disease with nutritional risk factors rather than a primary dietary calcium deficiency. Principal non-nutritional differentials: for skeletal deformity and pathological fracture, congenital and hereditary osteochondrodysplasias, osteogenesis imperfecta, chronic kidney disease with renal secondary hyperparathyroidism, hyperadrenocorticism, primary hyperparathyroidism, neoplasia, and trauma; for stiffness and shifting lameness, infectious and immune-mediated polyarthritis, osteoarthritis, laminitis, and toxic myopathy; for periparturient recumbency, hypomagnesaemia, hypophosphataemia, ketosis, toxic mastitis or metritis, obturator paralysis, and traumatic injury. Confirmatory tests: total and ionized calcium, phosphorus, magnesium, alkaline phosphatase, parathyroid hormone, 25-hydroxyvitamin D, renal biochemistry and urinalysis, and diagnostic imaging; for enzootic calcinosis, documented access to calcinogenic plants together with histopathological confirmation of metastatic calcification. Abbreviations: Ca, calcium; P, phosphorus; PTH, parathyroid hormone; Mn, manganese; DCAD, dietary cation–anion difference.

#### 3.2.4. Musculoskeletal System—Muscle Mass, Joint Function and Neuromuscular Pathologies

Evaluation of the musculoskeletal system requires differentiating adipose-tissue reserves (assessed via BCS) from skeletal muscle mass. Muscle wasting occurs via two principal metabolic pathways: sarcopenia (age-related loss of lean mass occurring in the absence of underlying disease or hypovitaminosis D) and cachexia (cytokine-mediated muscle loss driven by underlying chronic inflammatory, renal, or cardiac disease) [8,9]. The Muscle Condition Score (MCS) uses visual assessment and palpation across the temporal bones, scapulae, lumbar vertebrae, and pelvic bones to detect muscle trophism even in overweight or obese patients [8,9,10]. In research studies, MCS showed substantial intra-rater repeatability but only moderate inter-rater reproducibility: overall κ for inter-rater agreement was 0.50 in dogs, with intra-rater κ ranging from 0.59 to 0.77, and 0.43 in cats, with intra-rater κ from 0.49 to 0.76 [8,9]. Agreement was highest for animals with normal muscle mass or severe muscle loss and lowest in the mild-to-moderate range, which is precisely the range of greatest clinical interest [8,9]. Convergent validity against dual-energy X-ray absorptiometry, quantitative magnetic resonance, and ultrasonographic measurement is nonetheless established [8,9,10]. Two operational consequences follow. First, MCS is a screening instrument whose reproducibility is lowest in early muscle wasting, and its findings therefore require corroboration by additional tissue-level and laboratory evidence. Second, MCS has been validated in dogs and cats only at present; extrapolation to other species is not currently supported, and species-adapted instruments, such as the equine Muscle Condition Score, must be used where available.

Selenium and Vitamin E Deficiency (Nutritional Myodegeneration/White Muscle Disease): Selenium (as a cofactor of glutathione peroxidase) and vitamin E (α-tocopherol) act as synergistic cellular antioxidants protecting myocyte membranes against reactive oxygen species and lipid peroxidation. Deficiency leads to acute nutritional myodegeneration (white muscle disease), presenting as muscle stiffness, weakness, myocardial failure, and bilateral skeletal-muscle necrosis, documented as a case series in foals [34] and classically described in rapidly growing calves, lambs, and kids, in which bilaterally symmetrical skeletal and cardiac myodegeneration follows maternal selenium deficiency during gestation [35,36]. In lambs, the cardiac form has been characterized by marked elevation of cardiac troponin I and of the myocardial band of creatine kinase, which distinguish acute myocardial involvement from the subacute skeletal presentation [37]. The same syndrome is reported in camelid crias [35]. In pigs, deficiency triggers dietetic hepatic necrosis and mulberry heart disease. Confirmation requires whole-blood or serum selenium and erythrocyte glutathione peroxidase activity, plasma α-tocopherol, and serum creatine kinase, together with exclusion of exertional, traumatic, toxic (notably ionophore) and infectious myopathies [35].

Vitamin E Deficiency and Equine Motor Neuron Disease (EMND): In horses, prolonged somatic vitamin E deficiency leads to selective oxidative destruction of lower motor neurons within the ventral horns of the spinal cord and brainstem [38]. EMND presents clinically with progressive neurogenic muscle atrophy, muscle fasciculations, marked weight loss despite a normal appetite, and a characteristic posture with all four feet tucked under the body, frequent weight-shifting, and low head carriage [38]. EMND is a horse-specific entity and must not be treated as a general marker of vitamin E deficiency across species: the same nutrient deficit produces nutritional myodegeneration in ruminant and equine neonates, and neuroaxonal or retinal lesions in other contexts. Confirmation requires plasma α-tocopherol together with ventral branch of the spinal accessory nerve or sacrocaudalis dorsalis medialis muscle biopsy [38]. Reference intervals for plasma α-tocopherol must be interpreted against the management system, since free-ranging horses on natural pasture may show markedly different vitamin E status from stabled animals on preserved forage [39]. Marginal α-tocopherol status is also not a silent finding outside the neuromuscular system: in breeding stallions, dietary supplementation with Zn/Se and α-tocopherol modified the metabolic milieu, the haemogram, and semen traits, indicating that the consequences of marginal vitamin E and trace-element status extend to erythrocyte stability and reproductive performance, and should therefore be sought in the reproductive and haematological history as well as in the muscle examination [40].

Feline Diabetic Polyneuropathy (Plantigrade Stance/”Low Heels”)—a nutrition-related metabolic disease, not a primary nutrient deficiency (Category B): Chronic, unmanaged hyperglycaemia in diabetic cats induces a distal, symmetrical, metabolic polyneuropathy characterized by Schwann-cell dysfunction, axonal degeneration, and impaired axonal transport in the sciatic nerve [41,42]. Clinically, this manifests as progressive hindlimb weakness, loss of patellar reflexes, muscle wasting, and a characteristic plantigrade stance (“low heels”, or hock-dropping), whereby the cat is supported by the hocks rather than the digits [41,42]. The entity is not caused by a nutrient deficiency but is included here on two operational grounds: it is a visually unmistakable, bedside-detectable sign of a metabolic disease whose long-term management is substantially dietary, and it is the principal differential for the plantigrade posture of manganese-dependent perosis described above, from which it must be distinguished during clinical examination.

Joint Function and Cartilage Matrix—nutraceutical evidence, not deficiency semiotics (Category D): This item is included as a decision aid at the point of intervention, not as a diagnostic sign, and no nutrient deficiency state corresponds to it. A 2022 systematic review and meta-analysis of 57 articles comprising 72 trials reported good evidence of clinical benefit for ω-3-enriched diets in canine and feline osteoarthritis, attributed to competition of EPA and DHA with arachidonic acid for cyclo-oxygenase and lipoxygenase, whereas oral chondroitin sulfate and glucosamine did not demonstrate significant analgesic or anti-inflammatory efficacy over a placebo [43]. The implication for the assessment is limited but concrete: when joint disease is identified during the musculoskeletal examination, dietary ω-3 provision should be quantified as part of the nutritional plan.

**Table 4:** Synoptic screening table for nutritionally relevant neuromuscular, muscular, and joint alterations.

Nutrient/Condition	Clinical Sign/Lesion	Mechanism (Species Specific)	Literature
Selenium/Vitamin E (Deficiency)	Nutritional myodegeneration (“white muscle disease”), muscle weakness	Lipid peroxidation of myocyte membranes; calves/lambs and foals (WMD); pigs (mulberry heart disease and hepatic necrosis)	Delesalle et al., 2017 [34]
Vitamin E (Chronic Deficiency in Equines)	Equine motor neuron disease (EMND), severe muscle wasting	Oxidative degeneration of lower motor neurons (ventral horns); neurogenic muscle atrophy and “tucked-in” posture in horses	Divers et al., 1994 [38]
Chronic Hyperglycaemia (Diabetes Mellitus)	Diabetic polyneuropathy, plantigrade stance (“low heels”), hindlimb weakness	Sciatic axonal degeneration and demyelination secondary to chronic hyperglycaemia; specific to feline diabetes mellitus	Mizisin et al., 2002 [41]; Mizisin et al., 2007 [42]
Omega-3 Fatty Acids (EPA/DHA)	Joint-pain reduction, improved mobility	Inhibition of the pro-inflammatory eicosanoid cascade (PGE2); evidence of clinical benefit in canine and feline osteoarthritis (systematic review and meta-analysis)	Barbeau-Grégoire et al., 2022 [43]
Chondroitin/Glucosamine [D]	No primary deficiency sign; efficacy not demonstrated	Lack of clinical analgesic efficacy demonstrated in a systematic meta-analysis in dogs and cats	Barbeau-Grégoire et al., 2022 [43]

Footnotes to Table 4. Category codes: selenium and vitamin E deficiency, and chronic equine vitamin E deficiency (EMND), are Category A; chronic hyperglycaemia with diabetic polyneuropathy is Category B, a metabolic disease that is nutritionally managed but not nutritionally caused; ω-3 fatty acids and chondroitin/glucosamine are Category D, entered as intervention-evidence rows rather than as deficiency states, and no deficiency syndrome corresponds to chondroitin or glucosamine. Principal non-nutritional differentials: for generalized weakness and muscle atrophy, disuse, cachexia of chronic inflammatory, renal, cardiac or neoplastic disease, endocrinopathy (hypothyroidism, hyperadrenocorticism, or hyperthyroidism in cats), immune-mediated and infectious myositis, exertional and toxic myopathies including ionophore toxicosis, inherited myopathies, and peripheral neuropathies of non-metabolic origin; for plantigrade stance specifically, tarsal or Achilles mechanism injury, lumbosacral disease, and polyneuropathy of other cause. Confirmatory tests: serum creatine kinase and aspartate aminotransferase, whole-blood or serum selenium with erythrocyte glutathione peroxidase, plasma α-tocopherol, serum fructosamine and blood glucose, electrodiagnostic studies, and muscle or nerve biopsy. Abbreviations: MCS, Muscle Condition Score; EMND, equine motor neuron disease; EPA, eicosapentaenoic acid; DHA, docosahexaenoic acid; PGE2, prostaglandin E2.

#### 3.2.5. Metabolic and Endocrine Homeostasis—Choline Dynamics, Leptin Resistance, and Energy-Substrate Partitioning

Systemic energy balance and lipid distribution involve tightly regulated endocrine, hepatic, and metabolic cross-talk. While L-carnitine facilitates the translocation of long-chain fatty acids across the inner mitochondrial membrane for β-oxidation [44,45], choline serves an essential, complementary role in hepatic lipid mobilization. As an obligatory precursor for phosphatidylcholine (lecithin), choline is required for the assembly and secretion of very-low-density lipoproteins (VLDLs) from hepatocytes [46]. In cats, choline availability is one determinant of hepatic VLDL export, and impaired export contributes to intracellular triglyceride accumulation [44,46]. Feline hepatic lipidosis (FHL) is, however, a multifactorial syndrome and not a choline-deficiency disease: the usual precipitant is a period of anorexia or rapid weight loss in a previously obese cat, upon which insulin resistance, massive peripheral lipid mobilization, and relative deficits of choline, methionine, taurine, arginine, carnitine, and B vitamins are superimposed [46]. It is accordingly classified as a nutrition-related metabolic disease (Category B) with a strong management component, and the clinical priority is the identification and reversal of the anorexia rather than the correction of any single nutrient. The equid counterpart of this syndrome is hyperlipaemia, to which donkeys are particularly prone, and it follows the same logic: a period of negative energy balance in an animal with substantial fat reserves triggers massive triglyceride mobilization and hepatic lipid accumulation. A recent clinical report illustrates how the assessment sequence proposed here operates in practice, the diagnosis in an emaciated jenny resting on the combination of Body Condition Score, Muscle Condition Score, and faecal score at presentation, with nutritional rehabilitation planned accordingly and refeeding syndrome anticipated [47].

Leptin Resistance and Equine Metabolic Syndrome (EMS): Leptin is an adipocyte-derived hormone whose circulating concentration reflects total adipose mass; under physiological conditions, it binds hypothalamic receptors to suppress appetite and elevate basal energy expenditure. Sustained obesity, however, induces central and peripheral leptin resistance, whereby high circulating leptin levels fail to suppress hyperphagia. In equids, hyperleptinaemia and leptin resistance are features of equine metabolic syndrome (EMS), a nutrition-related endocrine-metabolic disorder rather than a nutrient deficiency, whose central abnormality is insulin dysregulation and whose principal clinical consequence is endocrinopathic laminitis [48]. EMS is classified here as Category B: diet and body condition are the dominant modifiable risk factors and the mainstay of control, but the diagnosis rests on dynamic insulin testing and not on nutritional semiotics.

Interspecies Adipokine Profiles and Inflammatory Signalling: Adipose tissue operates as a dynamic endocrine organ producing adipokines, primarily adiponectin, leptin, and pro-inflammatory cytokines such as TNF-α and IL-6. Adiponectin exerts anti-inflammatory and insulin-sensitizing effects, and its serum concentration is inversely correlated with body-fat percentage [49]. Leptin, moreover, is not exclusively of adipose origin and is not solely a signal of energy stores: in cats, it is produced locally and its receptor expressed within ovarian tissue, with the pattern varying according to reproductive phase even in underweight queens [50]. This local, tissue-level activity is a reminder that a circulating adipokine concentration cannot be read as a simple index of body fat, and it further limits the diagnostic use of these analytes in nutritional assessment. In dogs and cats, obesity is characterized by marked systemic elevations of TNF-α and IL-6, driving chronic low-grade inflammation; conversely, in horses, obesity is associated with suppression of circulating adiponectin, in turn associated with vascular endothelial dysfunction and tissue insulin resistance [48,49]. Adipokine measurement is not, at present, a routine clinical test in most practices, and no adipokine threshold is proposed here as a decision rule. The material is included because it explains why obesity carries different downstream consequences in different species, which bears directly on the interpretation of a high BCS; the assays themselves are assigned to the specialist and confirmatory tier defined below.

Energy-Substrate Partitioning—Monogastrics (Dogs, Cats, and Pigs): Monogastric species depend primarily on the enzymatic digestion and absorption of exogenous carbohydrates, but fundamental enzymatic and regulatory differences exist between omnivores/facultative carnivores and obligate carnivores. Swine and dogs express active hepatic glucokinase (hexokinase IV), which dynamically adapts to dietary carbohydrate intake and down-regulates gluconeogenic enzymes when dietary glucose is abundant [51]. Domestic cats, in contrast, possess minimal or absent hepatic glucokinase activity, relying on low-K_m_ hexokinases that saturate at low glucose concentrations, and lack adaptive transcriptional regulation of hepatic transaminases (ALT and AST) and gluconeogenic enzymes, which remain constitutively active regardless of dietary composition [21,22,51]. Consequently, the question is how and to what extent cats use carbohydrates as a source of energy, where adequate protein amounts and specific essential amino acids are strictly required for gluconeogenesis, as a primary substrate for ATP production, along with other functional roles [21,22,51].

Trace-element supplementation trials illustrate the same caution: chromium supplementation did not significantly modify glucose tolerance in either obese or non-obese cats, despite a mechanistically plausible insulin-sensitizing rationale. The clinical fact generalizes well beyond trace minerals, for an intervention cannot be assumed to be clinically effective merely because its mechanism is plausible and must be validated empirically within each species and each body-condition state [52].

Foregut Fermenters (Cattle, Sheep, and Goats) and Hindgut Fermenters (Horses): These two groups are physiologically distinct and are grouped here solely with respect to the trait of fermentative energy supply; horses are hindgut fermenters and do not ruminate. Ruminants ferment in the reticulorumen, proximal to the small intestine, whereas equids ferment in the caecum and colon, distal to it, and retain functional small-intestinal starch digestion with a consequently greater glycaemic and insulinaemic response to cereal feeding. With that distinction established, both groups rely on microbial fermentation of complex dietary carbohydrates (cellulose and hemicellulose) into volatile fatty acids (VFAs)—predominantly acetate, propionate, and butyrate [53]. Minimal dietary glucose reaches the small intestine, and hepatic gluconeogenesis—using propionate as the principal substrate—is required continuously to maintain basal blood-glucose levels [53]. Peripheral tissues in ruminants and equids use acetate rather than glucose as the primary precursor for lipogenesis, resulting in lower baseline glycaemia and a hormonal regulation of nutrient storage distinct from that of monogastric species [53].

**Table 5:** Synoptic screening table for metabolic pathways, adipokine dysregulation, and energy-substrate utilization across species.

Metabolic Pathway/Factor	Clinical/Metabolic Phenotype	Mechanism (Species Specific)	Literature
Choline Deficiency vs. L-Carnitine	Feline hepatic lipidosis (FHL), hepatic steatosis, weight-loss resistance	Impaired phosphatidylcholine synthesis inhibits VLDL packaging and hepatic triglyceride export; critical in cats and dogs	Center et al., 2000 [44]; Center et al., 2012 [45]; Verbrugghe & Bakovic, 2013 [46]
Hyperleptinaemia and Leptin Resistance	Persistent hyperphagia, refractory obesity, endocrinopathic laminitis	Hypothalamic leptin-receptor desensitization; characteristic feature of equine metabolic syndrome (EMS) in horses/ponies	Frank et al., 2010 [48]
Adiponectin Suppression and Inflammatory Adipokines	Systemic insulin resistance, chronic vascular/systemic inflammation	Adiponectin drops during obesity; TNF-α/IL-6 elevated in dogs/cats; adiponectin severely suppressed in horses	Radin et al., 2009 [49]
Monogastric Glucokinase and Constitutive Gluconeogenesis	Obligate dietary-protein requirement for energy in cats; flexible carbohydrate utilization in dogs/pigs	Glucokinase present in pigs/dogs (dynamic enzyme regulation); glucokinase absent/minimal in cats (constitutive gluconeogenic transaminases requiring amino acids)	Verbrugghe & Hesta, 2017 [51]; Morris, 2002 [21]; Macdonald et al., 1984 [22]
VFA Fermentation vs. Direct Glucose Utilization	Lower resting glycaemia, reliance on continuous gluconeogenesis	Microflora produce VFAs (acetate, propionate, butyrate); propionate is the main gluconeogenic precursor in ruminants/equids	Aschenbach et al., 2011 [53]

Footnotes to Table 5. All rows in this table describe metabolic traits or nutrition-related metabolic diseases (Category B), with the exception of the interspecies substrate-partitioning rows, which describe normal physiology rather than pathology and are included to constrain cross-species inference. None of these entries is a primary nutrient deficiency. Principal non-nutritional differentials: for hepatic steatosis, diabetes mellitus, hyperadrenocorticism, hyperlipidaemia syndromes, toxic and infectious hepatopathies, and cholangitis; for obesity refractory to restriction, hypothyroidism, hyperadrenocorticism, insulinoma, and under-reported intake; for laminitis, pituitary pars intermedia dysfunction, sepsis-associated and supporting-limb laminitis. Confirmatory tests: hepatic ultrasonography with fine-needle aspirate or biopsy, serum bile acids, dynamic insulin testing (oral sugar test and insulin tolerance test), basal insulin and adrenocorticotropic hormone, and thyroid testing. Adipokine assays are specialist tests without established clinical decision thresholds in veterinary species and are not proposed here as diagnostic criteria. Abbreviations: VLDL, very-low-density lipoprotein; FHL, feline hepatic lipidosis; EMS, equine metabolic syndrome; TNF-α, tumour necrosis factor alpha; IL-6, interleukin 6; VFA, volatile fatty acid; ALT, alanine aminotransferase; AST, aspartate aminotransferase.

#### 3.2.6. Oral Cavity Pathology, Gastrointestinal Function, and Urinary Excretion Dynamics

The oral cavity, the gastrointestinal (GI) tract, and the renal/urinary excretory pathway constitute an integrated physiological axis governing nutrient acquisition, digestive processing, and metabolic-waste clearance. Pathological disruption at any point along this continuum directly impairs systemic nutritional homeostasis and energy balance.

Oral Cavity Pathology and Masticatory Efficiency—causes of reduced intake, not lesions of nutritional origin (Category C): Dental attrition, severe periodontal disease, feline tooth resorption, feline chronic gingivostomatitis, buccal mucosal ulceration, and equine dental malocclusions (e.g., enamel points and wave mouth) significantly compromise prehension and masticatory efficiency [1,54]. Severe oral pain leads to hyporexia, selective feeding behaviours, or rapid swallowing of unchewed food. Inadequate mechanical breakdown reduces salivary buffering and enzyme mixing, delaying gastric digestion and secondarily driving progressive protein-energy malnutrition regardless of dietary nutrient density [1,54]. Local periodontal inflammation also triggers translocation of pro-inflammatory cytokines (TNF-α, IL-1β, and IL-6) into systemic circulation, escalating systemic inflammatory load and exacerbating peripheral tissue catabolism [49,54]. Clinical evaluation by a veterinary dentistry specialist is strongly indicated to diagnose the underlying pathology and to restore oral health as part of a multidisciplinary approach; post-procedural dietary modification—such as transitioning to kibble with an engineered, cross-linked fibre matrix for mechanical plaque removal, high-palatability wet/canned textures, or liquid recovery diets—is critical to maintain voluntary caloric intake [1,54].

Gastrointestinal Digestive Efficiency and Faecal Scoring: Faecal quality is a practical, non-invasive proxy indicator of digestive function and belongs within the overall nutritional assessment. Faecal consistency, form, and colour provide a useful clinical signal, but the instrument must match the species. The seven-point faecal-scoring charts in common use were developed for, and are routinely applied in, dogs and cats [55,56]; they have no established equivalence in ruminants, equids, birds, or reptiles, in which normal output ranges from formed faecal balls to urate-containing droppings, and they must not be transposed to those *taxa*. Species-appropriate systems should be used instead, such as faecal consistency scoring in dairy cattle, or structured qualitative description referenced to the normal output for the species; in birds and reptiles, the assessment additionally includes the urate fraction, which has no mammalian counterpart. In every species, faecal score is a screening prompt and never, by itself, evidence of a nutrient imbalance. Intestinal transit time, nutrient-absorption efficiency, mucosal-barrier integrity, and gut-microbiota stability are indirectly estimated through faecal-score assessment [55,56]. Dietary physical form (extruded kibble, canned wet food, and coarse vs. finely ground forage), processing parameters, feeding schedule (time-restricted vs. ad libitum feeding), and fibre-matrix composition all influence digestive efficiency to differing extents across species. Balanced ratios of soluble/fermentable fibres (promoting short-chain fatty-acid production for colonocytes) and insoluble fibres (regulating transit time) optimize stool consistency and mucosal health, whereas abrupt dietary shifts, nutrient imbalance, or low-digestibility formulations disrupt digestive function, microbial balance, and nutrient absorption [56].

Feeding Environment and Behaviour: Environmental stressors, multi-pet competition for food bowls, feeding location, and anxiety disorders significantly modulate autonomic input to the GI tract. Stress-induced sympathetic overdrive can produce psychogenic abnormal eating behaviours (e.g., rapid gorging followed by regurgitation, stress-induced diarrhoea, or psychogenic anorexia) [57]. In such scenarios, environmental enrichment and spatial separation of feeding stations should be implemented alongside dietary adjustment [57]. Referral to a veterinary behaviour specialist is desirable where that resource exists. In group-housed livestock, the equivalent intervention is management of feed-bunk space, stocking density, and social hierarchy; in exotic species, it is enclosure design and the spatial and temporal distribution of feeding opportunities.

Urine Specific Gravity (USG) and Hydration Assessment: USG provides a practical, albeit indirect, index of renal concentrating activity, systemic hydration status, and voluntary daily water intake relative to metabolic demand [58,59]. Obligate carnivores (cats) possess an evolutionary adaptation for high renal concentrating ability, resulting in an elevated baseline USG (>1.035). Concentrated urine markedly elevates the relative supersaturation (RSS) of lithogenic crystals (calcium oxalate and struvite), increasing the risk of feline lower urinary tract disease (FLUTD) and urolithiasis [58,59]. High-moisture therapeutic diets (>75% moisture) increase overall daily water intake, expand total urine volume, and decrease USG, thereby reducing urinary solute concentration and lithogenic crystal-nucleation potential [58,59]. USG is nonetheless interpretable only in context: chronic kidney disease, diabetes mellitus and diabetes insipidus, hyperthyroidism, hyperadrenocorticism, hepatic insufficiency, pyometra, and treatment with diuretics, corticosteroids, or intravenous fluids all impair renal concentrating ability and will lower USG independently of water intake. A low USG must therefore prompt exclusion of these causes before it is read as evidence of adequate hydration or of dietary moisture adequacy [59].

Electrolyte Modulation (KCl vs. NaCl) and Relative Supersaturation: Regulating the dietary cation–anion balance (DCAB) maintains an optimal urinary pH to prevent either struvite (pH > 7.0) or calcium-oxalate (pH < 6.0) precipitation [58,59].

**Table 6:** Synoptic screening table for nutritionally relevant oral, gastrointestinal, and urinary parameters.

Parameter/Nutrient	Clinical Sign/Parameter	Mechanism (Species Specific)	Literature
Oral Pathology and Mastication [C]	Dental attrition, periodontal disease, FCGS, buccal ulcers, weight loss	Pain impairs prehension and mechanical reduction of food; systemic inflammatory cytokine release promotes tissue catabolism	Freeman et al., 2011 [1]; Niemiec, 2008 [54]
Faecal Scoring System and Dietary Form	Altered stool form, consistency, colour (1–7 score)	Non-invasive proxy for transit time, nutrient digestibility, and microflora balance; governed by dietary fibre ratios and feed processing	Guy et al., 2024 [55]; Cave et al., 2023 [56]
Feeding Environment and Behaviour [D]	Gorging/regurgitation, stress diarrhoea, psychogenic anorexia	Stress-induced autonomic GI dysmotility and inter-animal competition; managed via environmental modification and behavioural consultation	Overall, 1997 [57]
Water/Dietary Moisture	Urine specific gravity (USG) elevation (>1.035 in cats)	Indirect biomarker of fluid-intake adequacy; high-moisture diets (>75%) dilute urine and reduce crystal-nucleation risk	Bijsmans et al., 2021 [58]; Queau, 2019 [59]
Dietary Na/K Ratio (as KCl)	Reduced USG, lowered calcium-oxalate and struvite RSS	Promotes renal diuresis and urine dilution in dogs and cats while avoiding the sodium load associated with NaCl	Bijsmans et al., 2021 [58]

Footnotes to Table 6. Category codes: oral pathology and mastication are Category C, that is, diseases causing secondary malnutrition through impaired prehension and intake, and not lesions of nutritional origin; feeding environment and behaviour are Category D; faecal scoring and urine specific gravity are monitoring parameters rather than aetiological entries, and dietary sodium and potassium manipulation is a Category D intervention row. Principal non-nutritional differentials: for altered faecal quality, endoparasitism, infectious enteritis, chronic inflammatory enteropathy, exocrine pancreatic insufficiency, dysbiosis, intestinal neoplasia, and drug effects; for reduced intake, systemic disease of any organ system, pain, pyrexia, nausea, and neoplasia; for altered urine specific gravity, chronic kidney disease, diabetes mellitus and insipidus, hyperthyroidism, hyperadrenocorticism, hepatic insufficiency, pyometra, and diuretic, corticosteroid or fluid therapy. Confirmatory tests: full oral examination under general anaesthesia with dental radiography, faecal flotation and specific pathogen testing, trypsin-like immunoreactivity, cobalamin and folate, abdominal imaging, complete urinalysis with sediment and urine protein-to-creatinine ratio, and renal biochemistry. The 7-point faecal-scoring chart is established for dogs and cats only and must not be transposed to other taxa. Abbreviations: FCGS, feline chronic gingivostomatitis; USG, urine specific gravity; RSS, relative supersaturation; DCAB, dietary cation–anion balance; KCl, potassium chloride; NaCl, sodium chloride; GI, gastrointestinal.

### 3.3. Laboratory Evaluation, Serum Biochemical Biomarkers, and Haematological Profile

Physical and clinical nutritional findings, including BCS and MCS, should where possible be corroborated by objective laboratory data, with the essential qualification that almost every marker discussed below is non-specific and that none is diagnostic of a nutritional disorder when considered in isolation. In donkeys, the association of body fatness with the circulating metabolic profile has already been addressed for the breed concerned [60]. Two tiers are distinguished throughout. First-line tests, appropriate as a minimum database in general practice, comprise the complete blood count with erythrocyte indices, serum total protein with albumin and globulin fractions, and, in the relevant species and physiological states only, NEFA and β-hydroxybutyrate. Specialist or confirmatory tests, requiring referral, dedicated laboratories, or species-specific reference intervals, comprise plasma or erythrocyte fatty-acid profiling, adipokine assays, trace-element and vitamin quantification, and tissue biopsy. The tier assignment for each species is summarized in Table 8. A comprehensive nutritional assessment requires evaluating hepatic synthetic capacity, amino-acid equilibrium, adipose-tissue lipid mobilization, and erythropoietic functional integrity [11,12,39,61].

Serum Protein Fractions and Anabolic/Catabolic Equilibrium: Serum total protein consists primarily of albumin and globulin fractions, which serve as a direct biomarker of hepatic protein synthesis, dietary amino-acid bioavailability, and systemic inflammatory status [12]. Albumin, synthesized exclusively by hepatocytes, accounts for approximately 50–65% of total serum protein in domestic mammals and maintains intravascular colloid osmotic pressure. Owing to its relatively long biological half-life (~8–10 days in dogs and cats; ~12–20 days in large domestic animals), hypoalbuminaemia may reflect chronic protein-energy malnutrition or essential amino-acid deficiency, but only once the substantially more common non-nutritional causes have been excluded: systemic inflammation, since albumin is a negative acute-phase protein; hepatic synthetic failure; protein-losing enteropathy or nephropathy; exudative or extensive skin disease; haemorrhage; and dilution by fluid therapy or overhydration [12]. In practice, a nutritional interpretation of hypoalbuminaemia is a diagnosis of exclusion and must never be made on the albumin value alone. As a negative acute-phase protein, albumin synthesis is also down-regulated during systemic inflammation driven by pro-inflammatory cytokines (TNF-α, IL-1β, and IL-6). The albumin-to-globulin (A:G) ratio provides a sensitive index of overall metabolic equilibrium, capturing the balance between structural amino-acid anabolism and catabolic/inflammatory states: a decreased A:G ratio highlights suppressed hepatic albumin synthesis coupled with hyperglobulinaemia driven by chronic inflammatory cytokines, positive acute-phase proteins, or antigenic stimulation, whereas a preserved A:G ratio is consistent with, but does not confirm, structural protein stability, since proportionate losses in both fractions, as occur in haemorrhage or protein-losing enteropathy, leave the ratio unchanged [12].

Energy Balance and Lipid Mobilization Biomarkers (NEFA and BHB): During states of negative energy balance, when metabolic energy expenditure exceeds net dietary energy intake, the body mobilizes adipose lipid stores to maintain basal energy homeostasis [11]. Circulating non-esterified fatty acid (NEFA) concentrations measure the acute rate of adipose-tissue lipolysis mediated by hormone-sensitive lipase; markedly elevated NEFA reflects rapid fat mobilization, and when hepatic NEFA uptake exceeds the liver’s capacity for complete β-oxidation or VLDL export, excessive re-esterification leads to hepatic triglyceride accumulation and steatosis [11]. β-Hydroxybutyrate (BHB) is the predominant circulating ketone body generated via incomplete hepatic β-oxidation of mobilized fatty acids. In transition dairy cattle, elevated pre- and postpartum plasma NEFA and BHB concentrations are essential herd-level monitoring tools for detecting subclinical ketosis and predicting metabolic disorders (e.g., abomasal displacement, fatty-liver syndrome, and suppressed milk production) [11]. In small ruminants, severe negative energy balance during late gestation, particularly in animals carrying multiple foetuses, drives hyperketonaemia, culminating in pregnancy toxaemia [11]. The evidentiary basis for NEFA and BHB is, however, largely confined to these two settings. Herd-level thresholds have been derived and validated for transition dairy cattle, and the analogous application in periparturient small ruminants is well supported; no comparable validated cut-offs exist for dogs, cats, horses, pigs, or exotic species, in which these analytes may be measured but must be interpreted qualitatively and against the individual’s own baseline, never against bovine reference thresholds [11]. Equid data are, however, accumulating: serial monitoring of NEFA, β-hydroxybutyrate, urea, and liver enzymes in weaned foals from weaning to 18 months of age has been used to track energy balance and hepatic status across growth stages, indicating that these analytes are interpretable in growing equids provided the referent is the animal’s own serial pattern rather than a bovine cut-off [62].

Haematological Profile and Nutritional Anaemias: Complete blood count (CBC) parameters—specifically red blood cell (RBC) count, haemoglobin (HGB) concentration, and haematocrit (HCT)—provide vital baseline data on erythropoiesis, oxygen-carrying capacity, and fluid dynamics [39,61]. HCT and RBC mass reflect systemic hydration status (haemoconcentration secondary to dehydration vs. haemodilution) and circulating red-cell volume [39,61]. Specific nutritional deficiencies alter erythrocyte morphology through distinct mechanisms: microcytic, hypochromic anaemia (reduced mean corpuscular volume and mean corpuscular haemoglobin concentration) primarily points to iron or copper deficiency, or to pyridoxine (vitamin B6) deficiency, all of which impair haemoglobin synthesis and iron-transport pathways, whereas macrocytic, normochromic/hypochromic anaemia (elevated MCV) signifies cobalamin (vitamin B12) or folate deficiency, which impairs DNA synthesis and nuclear maturation during erythroid progenitor-cell division [39,61]. Haematological profiling allows clinicians to distinguish nutritional anaemias from non-nutritional aetiologies such as immune-mediated haemolysis or haemoparasitic infection (e.g., *Babesia* spp., *Anaplasma* spp., or *Mycoplasma haemofelis*) [39,61]. One caveat is of first practical importance. In adult animals fed a commercially complete diet, microcytic hypochromic anaemia is far more often the consequence of chronic external or gastrointestinal blood loss, whether from parasitism, ulceration, neoplasia, or urinary loss, than of true dietary iron deficiency, which in adults is rare. Chronic haemorrhage must therefore be excluded before iron deficiency is attributed to the diet; genuine nutritional iron deficiency is essentially a condition of milk-fed neonates, most characteristically piglets.

Essential Fatty Acid (EFA) Status—Plasma and Erythrocyte Lipid Profiling (ω-6 and ω-3 PUFAs): Polyunsaturated fatty acids (PUFAs) play pivotal roles in maintaining cell membrane integrity, regulating cutaneous water-barrier function, modulating immune responses, and governing inflammatory cascade pathways [63,64,65]. Omega-6 (ω-6) series (linoleic and arachidonic acids): Linoleic acid (LA; 18:2ω-6) is an essential dietary fatty acid across all mammalian species, and it is the direct precursor for epidermal acylceramides, which are vital for constructing the stratum corneum permeability barrier. Dietary LA deficiency manifests clinically as a dry, dull coat, diffuse scaling dermatosis, epidermal hyperkeratosis, and increased transepidermal water loss [63,65]. Arachidonic acid (AA; 20:4ω-6): Obligate carnivores such as domestic felids exhibit negligible or absent hepatic Δ6-desaturase and Δ8-desaturase activities, rendering them unable to synthesize adequate AA from LA [66]. Consequently, AA is an essential dietary requirement for cats. Deficiency leads to impaired platelet aggregation, hepatic lipid accumulation, and reproductive failure [63,65]. Omega-3 (ω-3) series (ALA, EPA, and DHA) and inflammatory modulation: α-Linolenic acid (ALA; 18:3ω-3), eicosapentaenoic acid (EPA; 20:5ω-3), and docosahexaenoic acid (DHA; 22:6ω-3): EPA and DHA incorporate into cell membrane phospholipids, competing with AA for cyclooxygenase (COX) and lipoxygenase (LOX) enzymes. Higher circulating EPA/DHA levels shift eicosanoid synthesis toward less inflammatory mediators (3-series prostaglandins and 5-series leukotrienes) and stimulate specialized pro-resolving mediators (resolvins and protectins) [64]. Vitamin E (α-tocopherol) is an integral antioxidant component of the erythrocyte membrane, and its depletion increases susceptibility to oxidative haemolysis; plasma α-tocopherol should therefore be interpreted together with the haematological profile [39]. Plasma and erythrocyte fatty-acid profiling is a specialist test that is not routinely available in most veterinary settings, requires species-specific reference intervals, and is influenced by recent dietary intake; it is therefore proposed here as a confirmatory investigation in selected refractory cases, not as a screening tool.

**Table 7:** Diagnostic biomarkers for protein status, energy balance, and haematological evaluation in nutritional assessment.

Diagnostic Marker	Clinical/Metabolic Phenotype	Mechanism (Species Specific)	Literature
Serum Albumin	Hypoalbuminaemia, peripheral oedema, reduced oncotic pressure	Impaired hepatic protein synthesis due to chronic essential amino-acid deficiency, liver failure, or negative acute-phase protein response	Kaneko et al., 2008 [12]
Albumin-to-Globulin (A:G) Ratio	Decreased A:G ratio (altered protein equilibrium and systemic inflammation)	Reflects catabolic state, inadequate dietary amino-acid intake, or acute/chronic inflammatory protein shifts	Kaneko et al., 2008 [12]
NEFA/BHB (β-Hydroxybutyrate)	Visceral/subcutaneous fat mobilization, ketosis, hepatic steatosis	Measures lipolysis rate (NEFA) and incomplete hepatic β-oxidation (BHB); herd transition monitoring in dairy cattle and pregnancy toxaemia in small ruminants	Ospina et al., 2013 [11]
RBC/HGB/HCT and Cell Indices	Microcytic or macrocytic anaemia, pale mucous membranes, altered viscosity	Assesses erythropoiesis and hydration; differentiates iron/copper deficiency (microcytic) or folate/B12 deficiency (macrocytic) from haemorrhage/haemoparasites	Cappai et al., 2020 [39]; Cappai et al., 2024 [61]
Essential Fatty Acid Profile (ω-6/ω-3, LA, AA, EPA, DHA)	Scaly dermatosis, dull coat, impaired wound healing, systemic inflammation	Disruption of epidermal permeability barrier (LA depletion); obligate feline requirement for AA due to absent $\Delta^{6}$-desaturase; ω-3 anti-inflammatory eicosanoid modulation	Bauer 2006 [63]; NRC 2006 [65]; Rivers et al. 1975 [66]; Calder 2015 [64]

Footnotes to Table 7. Tier assignment: serum albumin, the albumin-to-globulin ratio, and the complete blood count with erythrocyte indices are first-line tests appropriate as a minimum database in general practice; NEFA and β-hydroxybutyrate are first-line in transition dairy cattle and periparturient small ruminants only, and have no validated species-specific thresholds elsewhere; the essential fatty acid profile is a specialist confirmatory test requiring species-specific reference intervals and is not a screening tool. Every marker in this table is non-specific. Hypoalbuminaemia requires exclusion of systemic inflammation, hepatic failure, protein-losing enteropathy and nephropathy, exudative skin disease, haemorrhage, and haemodilution before any nutritional interpretation. A preserved albumin-to-globulin ratio does not exclude proportionate loss of both fractions. Microcytic hypochromic anaemia in an adult animal indicates chronic blood loss until proven otherwise; true dietary iron deficiency is essentially confined to milk-fed neonates. Abbreviations: A:G, albumin-to-globulin ratio; NEFA, non-esterified fatty acid; BHB, β-hydroxybutyrate; RBC, red blood cell; HGB, haemoglobin; HCT, haematocrit; LA, linoleic acid; AA, arachidonic acid; EPA, eicosapentaenoic acid; DHA, docosahexaenoic acid; PUFA, polyunsaturated fatty acid.

### 3.4. Control of Cross-Species Extrapolation

A key principle of this framework is that a physiological mechanism established in one species must not be extrapolated to another without species-specific evidence [51]. In monogastrics (swine/poultry), phytic acid binds divalent cations (Zn^2+^, Ca^2+^, Fe^2+^, and Mg^2+^), forming insoluble complexes that reduce mineral bioaccessibility and require exogenous phytase-enzyme supplementation. Direct extrapolation of this monogastric model to horses is not warranted, but the equine evidence is dose-dependent and is stated here in full. In mature horses fed alfalfa hay and pelleted concentrate, that is, at the phytate loads typical of practical equine rations, phytase supplementation produced no significant effect on phosphorus output or apparent phosphorus digestibility, and phytate disappearance averaged 93%, indicating that hindgut microbial phytase activity is normally sufficient [67]. Under a substantially higher phytate challenge, however, in which wheat and rice bran supplied 55–56% of dietary phosphorus as phytate, phytase supplementation did significantly increase apparent calcium digestibility, from 26.4% to 31.5%, while leaving phosphorus digestibility unchanged [68]. The two findings are not in conflict: the equine hindgut has ample capacity for the phytate loads of conventional diets, and a measurable benefit emerges only once that capacity is exceeded by a bran-rich ration. The clinically correct statement is therefore not that phytate is irrelevant in horses, but that it is normally compensated. Marginal zinc or calcium deficiency in a horse on a conventional cereal-based diet should not be attributed to phytate binding without diagnostic confirmation, whereas a genuinely bran-rich ration warrants closer attention to calcium adequacy [67,68]. The correct cross-species inference is accordingly neither wholesale transfer nor blanket denial, but a dose- and context-qualified statement.

### 3.5. Extension to Avian, Reptile, and Exotic Companion Mammal Considerations

Although most of the mechanistic evidence assembled above derives from the companion-mammal and livestock literature, the underlying principle of tissue-specific nutritional semiotics extends to avian, reptilian, and small exotic mammal practice, provided that species-specific physiology is respected.

Psittacine and passerine birds: Beyond the manganese-dependent perosis described above [33], seed-predominant diets low in preformed vitamin A are a recognized precipitating factor for hypovitaminosis A-related squamous metaplasia of the oral, choanal, and respiratory epithelium in companion psittacines; targeted species-specific validation studies remain comparatively scarce and represent a priority area for future research.

Reptiles: Heliothermic and diurnal reptile species depend on UV-B-driven cutaneous synthesis of vitamin D3 from 7-dehydrocholesterol, analogous to the pathway described for ruminants and swine, and to a far more limited extent for horses [26]. Combined UV-B deficiency and an inverted dietary Ca:P ratio is the principal driver of nutritional secondary hyperparathyroidism (“metabolic bone disease”), clinically presenting as mandibular/maxillary softening (“rubber jaw”), long-bone bowing, and pathological fractures; the tissue-by-tissue logic set out above (integument → bone → laboratory profile) applies directly, although reptile-specific reference intervals must be used for laboratory corroboration.

Lagomorphs and guinea pigs: Rabbits are obligate hindgut fermenters that depend on caecotrophy (re-ingestion of caecotrophs) for microbial B-vitamin and protein recycling, so interruption of this behaviour (e.g., through obesity, dental disease, or orthopaedic pain) produces a secondary, behaviourally mediated nutritional deficiency that is captured by the oral-pathology and behavioural nodes. Guinea pigs share primates’ obligate dietary vitamin C requirements [23], so scorbutic mucosal and joint signs should be specifically screened for in this species.

These extensions are explicitly a proof of concept. The underlying evidence base derives overwhelmingly from dogs, cats, horses, and ruminants; for psittacines, reptiles, and small exotic mammals, the present section demonstrates only that the tissue-by-tissue logic is transferable in principle. Species-specific reference intervals, species-specific faecal and body-condition instruments, and dedicated validation studies are all presently lacking; structured observational case series contributed by clinicians, in cooperation with academic research groups, would be the most realistic means of generating the missing evidence. This section should not be used as a standalone diagnostic resource for those *taxa*, and its development into a usable species-specific guide is the single highest priority for future work.

### 3.6. Consolidated Operational Checklist

The preceding sections are summarized in Table 8 as a sequential working checklist. It is deliberately structured as a prompt list rather than as a numerical score, since no weighting of items has been carried out. The checklist specifies what to record, the minimum laboratory database appropriate at that step, and the threshold at which referral should be considered. Where a cut-off derives from a published and species-specific instrument, it is referenced accordingly, as for the nine-point BCS and four-point MCS charts in dogs and cats [1,3,8,9,10] and for the clinically accepted threshold of 5% unintentional loss of body mass. All remaining values, including every reassessment interval, are provisional clinical prompts rather than validated decision thresholds, and require adaptation to species, life stage, physiological status, and concurrent disease. Reassessment intervals are anchored to the turnover time of the tissue under review: a hair-cycle interval of six to eight weeks is a reasonable first approximation for the haired integument of dogs, cats, and horses, but it has no general validity and must not be transferred unmodified to the moult cycle of birds, to the ecdysis cycle of reptiles, to keratinized structures such as hooves, claws, and beaks, in which visible growth requires months, or to livestock species whose production cycle dictates the practicable examination interval. Reassessment should therefore be scheduled according to the documented turnover time of the target tissue in the species concerned. The intervals given in Table 8 apply to the small-animal and equine patient in the absence of species-specific data.

**Table 8:** Proposed operational checklist for the interspecies nutritional examination, with minimum laboratory database, referral triggers, and reassessment intervals.

Step/Domain (Provisional Prompts)	What to Record or Examine	Minimum Laboratory Database	Referral Trigger
1. Signalment and diet history	Species, breed, age, sex, reproductive/production status; diet brand and form; measuring device used; meals/day; treats, chews, table foods; diet change in last 3 months; medication and supplement history; water source and access	None at this stage	Home-prepared or unbalanced diet fed >4 weeks; any growing, gestating or lactating animal on a non-complete ration
2. BCS and MCS	BCS on the validated nine-point (dog/cat) or species-specific scale; MCS on the four-point scale in dogs and cats, or a species-adapted score where available; body weight and trend	None at this stage	BCS ≤ 3 or ≥8/9; MCS ≤ 2 with normal or high BCS; unintentional loss > 5% body weight
3. Integument and adnexa	Coat/plumage coverage, symmetry of alopecia, lustre, pigment; skin thickness, elasticity, scaling, seborrhoea, follicular hyperkeratosis; hoof, claw, or footpad integrity	Skin scrapings, cytology, fungal culture/PCR before any nutritional attribution	Lesions persisting after exclusion of parasitic, infectious, allergic and endocrine causes
4. Mucosae and eye	Oral, conjunctival and vaginal mucous membrane colour; ocular surface moisture, keratinization; night vision by history	CBC with erythrocyte indices; faecal occult blood if pallor present	Pallor with normal diet; any ocular surface change
5. Bone, muscle and joint	Skeletal proportion and symmetry relative to age and growth rate; palpable epiphyseal or periosteal change; muscle bulk at temporal, scapular, lumbar and pelvic sites; joint effusion, range of motion, gait, and posture	Total and ionized calcium, phosphorus, alkaline phosphatase, creatine kinase; selenium and α-tocopherol where myodegeneration is suspected	Pathological fracture; recumbency; progressive neurogenic atrophy; suspected metabolic bone disease
6. Oral cavity and GI output	Conscious oral examination; dental attrition, periodontal disease, resorptive lesions, ulceration, and malocclusion; faecal score using a species-appropriate system; vomiting, regurgitation, and borborygmi	Faecal flotation; cobalamin and folate, and trypsin-like immunoreactivity, where chronic enteropathy is suspected	Any oral pain or lesion (dental referral); chronic diarrhoea > 3 weeks
7. Urinary output and hydration	Voluntary water intake against species requirement; urine specific gravity; pollakiuria, stranguria, and haematuria	Complete urinalysis with sediment; renal biochemistry if USG is low	USG persistently low with exclusion of increased intake; crystalluria or urolithiasis
8. Metabolic profile	Energy balance in the relevant physiological window (transition, late gestation, lactation, or rapid weight loss)	Albumin and globulin with A:G ratio; NEFA and BHB in transition cattle and periparturient small ruminants only	Hypoalbuminaemia after exclusion of inflammation, hepatic and protein-losing disease
9. Synthesis and category	Assign each finding to Category A, B, C or D; state the confirmatory test; formulate the nutritional plan	As dictated by category	Any Category A diagnosis requiring diet reformulation—refer to veterinary nutritionist
10. Reassessment	Re-examine the affected tissue and re-score BCS/MCS	—	Provisional intervals, requiring species- and life-stage-specific adaptation. Haired integument and coat (dog, cat, and horse): 6–8 weeks, approximating one hair-cycle interval; not transferable to avian moult, reptilian ecdysis, or keratinized structures (hooves, claws, or beak), for which months are required. Muscle mass and body condition: 4 weeks. Metabolic and laboratory markers: 2–4 weeks. Growing animals: 2–4 weeks.

## 4. Discussion

The framework presented herein proposes a structured sequence for evaluating animal nourishment in clinical practice [2]. By integrating physical-examination techniques with comparative-nutrition principles, this framework addresses the diagnostic limitations of relying solely on BCS [1].

The individual clinical signs assembled here, such as achromotrichia, xerophthalmia, perosis, or white muscle disease, are each long established within specialized sub-disciplinary literature. What is proposed as new is the ordering of these dispersed findings into a single, tissue-sequenced examination workflow that crosses *taxa* and that is explicitly indexed to species-specific metabolic constraints, together with the classification of every entry by aetiological category. Previous works [6,7] report an in-depth evaluation confined to ruminants, including instruments that have no counterpart here, such as rumen fill scoring. The present work deliberately does not pursue that degree of species-specific depth, since transferability across taxa and the explicit control of cross-species extrapolation would not otherwise be feasible. Whether that organization delivers measurable clinical benefit over the existing guidelines is an empirical question that this review does not answer, and that the validation programme outlined below is designed to address [1,2,3]. A corollary of this position is that feeding is not synonymous with nourishing, since fatness reflects principally energy provision, and at times hormonal imbalance, rather than the adequacy of key nutrients within examinable tissues and organs. The holistic approach proposed here, accounting for interspecific differences and requirements, is intended to orient nutritional assessment in veterinary clinical practice towards a broader, scientifically updated and evidence-informed methodology, subject to the staged validation set out below.

This approach mirrors developments in human clinical dietetics, where the analogous NFPE competency tool progressed from a conceptual proposal to a formally validated instrument through sequential item-generation, Delphi-based content and face validation, and inter-rater reliability testing among registered dietitians [2,4,5]. That validation pathway offers a directly transferable template for the framework proposed here.

Furthermore, the framework emphasizes the diagnostic risk of uncritical cross-species extrapolation. As demonstrated by the differential metabolic handling of phytate in horses versus swine [67], and by the distinct synthetic capacities for vitamin C, vitamin D3, and niacin across taxa [23,26,29], comparative, species-specific physiological requirements must guide the clinician’s diagnostic reasoning rather than assumptions transferred wholesale from a better-studied reference species. Formulation of specific diets should therefore be undertaken by veterinary nutritionists in order to safeguard patient health, both under physiological conditions and when disease is suspected or confirmed. Interdisciplinary collaboration between clinicians and specialists is therefore strongly encouraged.

The present work was conceived to organize comparative-nutrition knowledge into a structured, tissue-related examination sequence, in a manner analogous to the way the NFPE extended general nutrition-risk screening in human dietetics into a physical-examination-based instrument [2]. An overarching principle governs the framework as a whole: mechanistic plausibility is not evidence of clinical effect. A nutrient with a well-characterized biochemical role in a pathway may nonetheless fail to alter any clinical endpoint when supplemented, and a binding interaction demonstrated in one species may be fully compensated in another. The framework is therefore constructed to move from sign to confirmatory test, rather than from mechanism to intervention. Finally, the strength of the evidence behind the table entries is not uniform. The entries range from randomized controlled trials and systematic reviews, through case series and cross-sectional studies, to standard reference texts and, in one instance, a historical report retained for priority only. The species in which each finding is documented, the aetiological category, the principal non-nutritional differentials, and the confirmatory test required are stated in the notes accompanying each table, so that the clinician may judge the evidential weight of each row directly, in the light of the individual clinical case.

It is nonetheless necessary to underline that the present framework should not be considered as a validated clinical protocol. Moreover, the review methodology, although structured, is narrative and purposive. Sources were selected for clinical relevance rather than by exhaustive enumerative screening. Clinicians should treat the tables as a curated clinical index rather than as an exhaustive or bias-free synthesis, and any future attempt to convert this framework into a formal clinical guideline would require the synthesis to be repeated under systematic-review conditions. Last but not least, the evidence base is markedly uneven across species: it is strongest for dogs and cats, strong for horses and ruminants, moderate for swine and poultry, but still weak for psittacines, reptiles, lagomorphs, and small exotic mammals. The framework should not be presented to practitioners as equally applicable across all *species.* MCS shows only moderate inter-rater reproducibility (κ 0.50 in dogs, 0.43 in cats), with weakest agreement precisely in the mild-to-moderate wasting range; BCS is validated principally in dogs and cats; the seven-point faecal-scoring chart has no established equivalent outside small animals; and most of the laboratory markers discussed are non-specific. Many of the clinical signs indexed in Table 1, Table 2, Table 3, Table 4, Table 5, Table 6 and Table 7 are non-specific. The tables are screening prompts that generate hypotheses; they are not diagnostic criteria, and their misuse as such would risk attributing to nutrition a sign whose cause may be parasitic, infectious, endocrine, inflammatory, toxic, or neoplastic, and which therefore requires differential diagnosis. The non-nutritional differentials and confirmatory tests stated in the table footnotes are intended precisely to prevent that error. A validation programme follows directly from the human NFPE precedent [2,4,5] and is proposed in four stages. Stage 1: item generation and reduction, refining the tissue-by-tissue items into a defined instrument with explicit operational definitions for each sign. Stage 2: content and face validation through a Delphi consensus of board-certified veterinary nutritionists, internal medicine specialists, and species specialists, conducted separately for companion-animal, equine, farm-animal, and exotic-species panels, since the item sets will legitimately diverge. Stage 3: reliability testing, with inter-rater and intra-rater agreement measured across clinicians of differing experience in each target species, using the same κ-based methodology applied to MCS [8,9]. Stage 4: criterion and predictive validation in prospective clinical cohorts, testing the instrument against objective nutrient status measures and, ultimately, against clinically meaningful outcomes. Only after Stage 3 would the framework merit description as an instrument; only after Stage 4 would it merit description as a validated one. In the interim, its legitimate function is as a structured support.

## 5. Conclusions

This paper proposes an interspecies, tissue-by-tissue framework for screening animal nourishment, intended for use alongside body and muscle condition scoring and within the existing WSAVA and AAHA nutritional assessment guidelines rather than in place of them. Its contribution is organizational, in that it assembles, to the best of the author’s knowledge for the first time in veterinary medicine, individually established comparative-nutrition findings into a single clinical sequence; it indexes that sequence to the species-specific metabolic constraints that make a given nutrient imbalance plausible, so that the sequence is interspecies rather than species-specific; it separates primary nutrient deficiencies and excesses from nutrition-related metabolic diseases, from diseases causing secondary malnutrition, and from management determinants of intake; and it states, for every entry, the non-nutritional differentials and the confirmatory test required. It is a proposal and not a validated instrument. Prospective assessment of diagnostic accuracy and species-specific refinement, most urgently for avian, reptile, and small exotic mammal patients, are all required before it can be recommended for routine unsupervised clinical use; the pathway followed by the human NFPE offers a concrete template for that programme. Until such data exist, its appropriate use is twofold: as a structured aide-mémoire that prompts the clinician to look beyond adiposity during an examination that is already being performed, and as a research agenda for veterinary clinical nutrition.

## Data Availability

No new data were generated in this study. The canine nutritional-assessment form referenced in the text is available from the corresponding author upon request.
